# Versatility of Biofilm Matrix Molecules in *Staphylococcus epidermidis* Clinical Isolates and Importance of Polysaccharide Intercellular Adhesin Expression during High Shear Stress

**DOI:** 10.1128/mSphere.00165-16

**Published:** 2016-10-05

**Authors:** Carolyn R. Schaeffer, Tra-My N. Hoang, Craig M. Sudbeck, Malik Alawi, Isaiah E. Tolo, D. Ashley Robinson, Alexander R. Horswill, Holger Rohde, Paul D. Fey

**Affiliations:** a Department of Pathology and Microbiology, Center for Staphylococcal Research, University of Nebraska Medical Center, Omaha, Nebraska, USA; b Bioinformatics Service Facility, Universitätsklinikum Hamburg-Eppendorf, Hamburg, Germany; c Department of Microbiology and Immunology, University of Mississippi Medical Center, Jackson, Mississippi, USA; d Department of Microbiology, University of Iowa, Iowa City, Iowa, USA; e Institut für Medizinische Mikrobiologie, Virologie und Hygiene, Universitätsklinikum Hamburg-Eppendorf, Hamburg, Germany; University of Kentucky

**Keywords:** accumulation-associated protein, *Staphylococcus epidermidis*, biofilms, catheter-associated infections, polysaccharide intercellular adhesin

## Abstract

*Staphylococcus epidermidis* is a leading cause of hospital-associated infections, including those of intravascular catheters, cerebrospinal fluid shunts, and orthopedic implants. Multiple biofilm matrix molecules with heterogeneous characteristics have been identified, including proteinaceous, polysaccharide, and nucleic acid factors. Two of the best-studied components in *S. epidermidis* include accumulation-associated protein (Aap) and polysaccharide intercellular adhesin (PIA), produced by the enzymatic products of the *icaADBC* operon. Biofilm composition varies by strain as well as environmental conditions, and strains producing PIA-mediated biofilms are more robust. Clinically, biofilm-mediated infections occur in a variety of anatomical sites with diverse physiological properties. To test the hypothesis that matrix composition exhibits niche specificity, biofilm-related genetic and physical properties were compared between *S. epidermidis* strains isolated from high-shear and low-shear environments. Among a collection of 105 clinical strains, significantly more isolates from high-shear environments carried the *icaADBC* operon than did those from low-shear settings (43.9% versus 22.9%, *P* < 0.05), while there was no significant difference in the presence of *aap* (77.2% versus 75.0%, *P* > 0.05). Additionally, a significantly greater number of high-shear isolates were capable of forming biofilm *in vitro* in a microtiter assay (82.5% versus 45.8%, *P* < 0.0001). However, even among high-shear clinical isolates, less than half contained the *icaADBC* locus; therefore, we selected for *ica*-negative variants with increased attachment to abiotic surfaces to examine PIA-independent biofilm mechanisms. Sequencing of selected variants identified substitutions capable of enhancing biofilm formation in multiple genes, further highlighting the heterogeneity of *S. epidermidis* biofilm molecules and mechanisms.

**IMPORTANCE** *Staphylococcus epidermidis* is a leading cause of infections related to biomaterials, mostly due to their ability to form biofilm. Biofilm accumulation mechanisms vary, including those that are dependent on specific proteins, environmental DNA (eDNA), or polysaccharide intercellular adhesin (PIA). We found that those isolates obtained from high-shear environments, such as the lumen of a catheter, are more likely to produce PIA-mediated biofilms than those isolates obtained from a low-shear biomaterial-related infection. This suggests that PIA functions as a mechanism that is protective against shear flow. Finally, we performed selection experiments documenting the heterogeneity of biofilm accumulation molecules that function in the absence of PIA, further documenting the biofilm-forming potential of *S. epidermidis*.

## Abstract (precis)

*Staphylococcus epidermidis* is a leading cause of infections related to biomaterials, mostly due to their ability to form biofilm. Biofilm accumulation mechanisms vary, including those that are dependent on specific proteins, environmental DNA (eDNA), or polysaccharide intercellular adhesin (PIA). We found that those isolates obtained from high-shear environments, such as the lumen of a catheter, are more likely to produce PIA-mediated biofilms than those isolates obtained from a low-shear biomaterial-related infection. This suggests that PIA functions as a mechanism that is protective against shear flow. Finally, we performed selection experiments documenting the heterogeneity of biofilm accumulation molecules that function in the absence of PIA, further documenting the biofilm-forming potential of *S. epidermidis*.

## INTRODUCTION

*Staphylococcus epidermidis* is among the most abundant species associated with the human skin microbiome (1, 2). Although its function in the context of this community is not well defined, its proximity to the epithelial layers facilitates colonization of indwelling medical devices, including intravascular catheters, orthopedic prosthetic devices, and cerebrospinal fluid (CSF) shunts (3). Due to this association with foreign bodies, *S. epidermidis* is the third most common cause of hospital-acquired infections and the number one cause of primary bacteremia (4). In contrast to *Staphylococcus aureus*, which produces an abundance of toxins, adherence factors, and cytolysins, the primary mechanism through which *S. epidermidis* evades the immune response is production of biofilm (5). Staphylococcal biofilms are multicellular aggregates surrounded by self-produced matrix materials that are functionally resistant to antibiotics (6) and components of the immune system (7). Therefore, current modalities to treat *S. epidermidis*-mediated biomaterial infections often necessitate removal of colonized devices and intensive antibiotic therapy, causing significant patient morbidity.

Recent studies of cellular organization within *S. aureus* biofilms have identified at least four distinct developmental steps, including adherence, multiplication, exodus, and maturation (8). Factors mediating *S. aureus* biofilm formation and/or structure, including adherence factors, extracellular DNA (eDNA) release, polysaccharide production, nuclease/protease activity, and phenol-soluble modulin factors, have been extensively studied and recently reviewed (9–13). Polysaccharide intercellular adhesin (PIA) was the first identified staphylococcal factor with a major function in biofilm accumulation (14–16). PIA is composed of repeating units of β-1,6-linked *N*-acetylglucosamine residues and is produced by the protein products of the *icaADBC* operon (14). Genetic regulation of *icaADBC*, and subsequent synthesis of PIA (or poly-*N*-acetyl glucosamine [PNAG], as it is referred to in *S. aureus* [17]), is governed by multiple direct and indirect transcriptional regulators, including IcaR, σ^B^, and SarA (18, 19). *icaADBC* appears to be ubiquitously present in *S. aureus* strains; however, recent evidence suggests that PIA/PNAG is preferentially expressed in methicillin-susceptible isolates of *S. aureus* (20–24) but repressed in those that exhibit methicillin resistance mediated by *mecA* (25). In contrast to *S. aureus*, not all strains of *S. epidermidis* carry the *icaADBC* operon. Several studies have indicated that *S. epidermidis* strains isolated from nosocomial environments or biomaterial-associated infections are more likely to carry *icaADBC* than those isolated from the skin of healthy individuals (26–31). In addition, among the strains that carry the *icaADBC* operon, some (e.g., strain 1457 [32, 33]) constitutively produce copious amounts of PIA and subsequent biofilm *in vitro*, whereas others such as CSF41498 (34) require salt or ethanol to induce *icaADBC* transcription and PIA synthesis.

Although many *S. epidermidis* strains causing biomaterial-related infections carry *icaADBC*, it is clear that protein-based matrices also have the ability to mediate biofilm accumulation and cause infection (30). In fact, certain strains have been shown to switch between PIA-dependent and protein-dependent biofilm matrices during prolonged infection (35). The two best studied among these biofilm proteins are accumulation-associated protein (Aap) and extracellular matrix binding protein (Embp) (36, 37). While these matrix components have unique structural characteristics, PIA-, Embp-, and Aap-dependent *S. epidermidis* biofilms are all resistant to phagocytosis due to their ability to inhibit NF-κB activation and interleukin-1β (IL-1β) production (38, 39). In general, staphylococcal biofilms skew the immune response, generating alternatively activated macrophages that promote a fibrotic rather than bactericidal response, thereby facilitating bacterial persistence (7, 40, 41).

In addition to the variety and variability of biofilm molecules, environmental conditions also affect biofilm properties (34, 38, 42–47). Though mechanistic details are lacking, multiple investigators have reported that PIA production is enhanced in fluid shear settings (42, 45), which may facilitate survival under certain *in vivo* growth conditions. For example, biofilm communities on a heart valve or central venous catheter (CVC) are subject to constant fluid movement and must be able to withstand high shear flow. Typical shear stress levels present in capillaries and venules range between 0.05 and 4 Pa, whereas levels in catheter lumens range between 0.02 and 3 Pa (45, 48, 49). In contrast, prosthetic joints and CSF shunts are exposed to minimal fluid shear at less-frequent intervals.

Numerous investigators have attempted to correlate the presence of *icaADBC* or *aap* with the virulence of isolates, yielding differing results (34, 47, 50–56). Many of these studies classify strains as either commensals/contaminants (isolated from skin) or infectious (isolated from blood or biomaterial). These analyses are complicated by the fact that most *S. epidermidis* infections are caused by the patient’s own flora (57–59). Examining “infectious” isolates as a whole may therefore yield highly variable and difficult-to-interpret results.

The heterogeneity of *S. epidermidis* strains, the variety of biofilm-mediated infections, and the knowledge that protein-dependent biofilms are less robust under shear flow conditions than those containing PIA (6) led us to hypothesize that *S. epidermidis* isolates from high-shear environments (e.g., CVCs) would more often carry *icaADBC* and produce more-robust PIA-dependent biofilm than would those from low-shear environments (e.g., prosthetic joints and CSF shunts). We addressed this and subsequent issues by examining strains isolated from clinical cases, grouped according to infection site. Additionally, we examined mutations that enhanced biofilm formation in an *icaADBC-*negative background.

## RESULTS

### Genetic diversity of clinical isolates.

**FIG 1:**
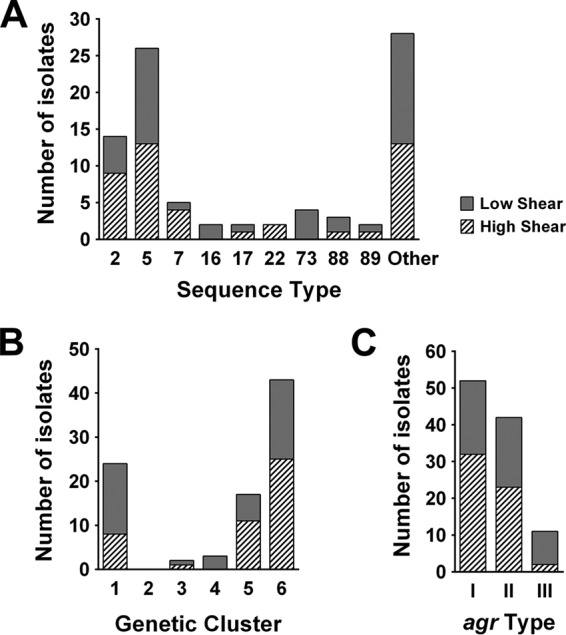
Population characteristics of clinical isolates. MLST was performed on 88 clinical isolates (low shear, *n* = 44; high shear, *n* = 44). (A and B) Distribution of clinical isolate sequence types (A) and genetic clusters (B) grouped by shear type. “Other” denotes STs represented by a single isolate. (C) *agr* type determined by PCR for 105 clinical isolates, grouped by shear type.

A total of 105 *S. epidermidis* patient isolates were retrospectively collected from Nebraska Medicine and placed into one of two groups according to the infection site. High-shear strains (*n* = 57) were isolated from blood cultures of patients with concomitant positive catheter tip cultures. Low-shear isolates (*n* = 48) were primarily from cases of orthopedic device failure necessitating explantation (*n* = 33) and cerebrospinal fluid shunt infections (*n* = 13). To test whether high- and low-shear populations were skewed toward specific genetic backgrounds, the sequence types (STs) of 88 isolates (44 from each shear group) were determined by multilocus sequence typing (MLST) and assigned to genetic clusters (GCs) (60, 61). The most common STs in both high- and low-shear populations were ST2 and ST5, although at least 50% of the strains in both shear groups belonged to STs other than ST2 and ST5 (Fig. 1A). GC analysis found that most isolates were identified as GC1, GC5, or GC6 (Fig. 1B); however, chi-square analysis demonstrated no statistical significance with respect to the results of comparisons of high- or low-shear isolates with specific GCs. These findings demonstrate that the clinical isolates examined in our study were from diverse backgrounds and provide further evidence that a wide variety of *S. epidermidis* strains are capable of causing disease.

Previous work demonstrated that the function of Aap varies based on proteolytic cleavage (37). Processing can occur via proteases produced by the host or the bacterium or both. In the staphylococci, the *agr* quorum sensing system regulates production of secreted proteases (62–65). Polymorphisms within the *agr* locus distinguish 3 *S. epidermidis* agr types (66, 67). To examine the possibility that specific *agr* types were associated with particular groups of clinical isolates, we determined the *agr* types of the clinical strains (Fig. 1C). *agr* type I was the most prevalent in both groups, representing 56% and 42% of the overall isolates from the high- and low-shear groups, respectively. A total of 40% of the isolates in both groups were type II. Only 11 isolates in total were *agr* type III; although there was a trend suggesting that *agr* type III was more extensively represented in the low-shear group (4% of the high-shear and 19% of the low-shear strains), more isolates would need to be assessed to determine the statistical significance of this observation. In conclusion, these studies demonstrated that there is not a significant difference in *agr* type between the high-shear and low-shear clinical isolate groups.

### The *ica* operon is associated with isolates from high-shear environments.

Genetic screening was used to test all isolates for genes responsible for production of 2 well-characterized biofilm matrix components: the *icaADBC* operon, which encodes enzymes that synthesize PIA; and *aap*, encoding Aap*.* Within the high-shear group (Table 1), 25 (43.9%) isolates contained *icaA* and 44 (77.2%) were positive for *aap*. Among the low-shear isolates (Table 2), 11 (22.9%) were *icaA* positive and 36 (75%) contained *aap.* Comparisons between shear groups revealed that significantly more isolates in the high-shear group carried the *icaADBC* operon (*P* < 0.05), while there was no significant difference between the groups with respect to the presence of *aap* (*P* > 0.05).

**TABLE 1:** High-shear clinical isolate characteristics^d^

Isolate ID	*aap*	Aap	*ica*	PIA	Biofilm^a^	*agr* type	ST^b^	GC^c^
4931	+	+	+	−	+	I	529	1
6540	+	+	−	ND	−	I	6	1
6591	+	+	−	ND	+	II	5	6
6609	+	+	−	ND	+	II	5	6
6638	−	ND	−	ND	+	I	533	1
6663	−	ND	+	+	+++	I	7	6
6829	+	+	+	+	+++	I	425	6
6850	−	ND	−	ND	+	II	ND	ND
6911	−	ND	−	ND	−	I	210	6
6973	+	+	+	−	+	I	2	5
7163	+	−	−	ND	+	II	17	6
7219	+	+	−	ND	+	II	5	6
7448	+	+	+	+	+++	I	7	6
7503	+	+	−	ND	+	II	5	6
7586	+	−	−	ND	+	II	5	6
7960	+	−	+	−	+	I	81	6
8116	+	+	+	−	+	I	2	5
8144	−	ND	+	+	++	II	535	6
8166	−	ND	−	ND	+	II	14	1
8183	+	+	−	ND	−	II	536	6
8246	−	ND	+	+	+++	I	22	5
8247	+	+	−	ND	−	III	88	1
8302	+	+	+	+	+	I	22	5
8481	+	+	+	+	+	I	2	5
8595	+	+	+	+	+++	II	ND	ND
8614	+	+	+	+	++	I	2	5
8635	+	+	−	ND	+	II	5	6
8889	+	+	+	+	+++	I	20	1
9193	+	+	+	−	+	I	2	5
9293	+	+	−	ND	+	III	ND	ND
9676	+	+	+	+	++	I	35	1
9797	+	+	+	−	+	I	2	5
10241	+	+	−	ND	−	I	5	6
10351	+	+	+	+	+	I	2	5
10546	+	+	−	ND	+	II	5	6
10935	+	+	−	ND	+	II	89	1
11038	+	+	+	+	+++	II	7	6
B4-11	+	+	−	ND	−	II	5	6
B14-11	−	ND	−	ND	−	II	5	6
B281-11	+	+	−	ND	+	I	2	5
B445-11	+	+	+	−	+	I	2	5
B460-11	+	+	+	+	+++	I	7	6
B503-11	+	+	−	ND	++	II	5	6
B658-11	+	+	−	ND	+	II	5	6
B818-11	−	ND	+	−	+	I	538	3
B922-11	+	+	−	ND	+	I	ND	ND
B931-11	+	+	−	ND	+	II	ND	ND
B935-11	+	+	−	ND	+	II	5	6
B983-11	+	+	−	ND	+	II	ND	ND
B1166-11	−	ND	−	ND	+	I	130	6
B1232-11	−	ND	−	ND	−	I	ND	ND
B1330-11	+	+	+	−	+	I	ND	ND
B1508-11	+	+	+	+	+++	II	ND	ND
B1528-11	+	−	−	ND	+	I	ND	ND
B1570-11	−	ND	−	ND	−	I	ND	ND
B1711-11	−	ND	−	ND	−	I	ND	ND
B1733-11	+	+	+	+	+++	I	ND	ND

a Biofilm formation compared to *S. epidermidis* 1457: –, <30%; +, 30% to 59%; ++, 60% to 84%; +++, ≥85%.

b ST, sequence type determined by MLST.

c GC, genetic cluster based on Bayesian analysis of population structure (BAPS) of MLST.

d ND, not determined. In columns 2 to 5, + indicates presence and − indicates absence.

**TABLE 2:** Low-shear clinical isolate characteristics^e^

Isolate ID	Source^a^	*aap*	Aap	*ica*	PIA	Biofilm^b^	*agr* type	ST^c^	GC^d^
4804	CSF	−	ND	+	−	++	I	23	5
4961	BF	+	+	−	ND	−	III	73	1
5018	BF	−	ND	−	ND	−	I	530	4
5183	BF	+	−	−	ND	−	II	531	3
5387	BF	+	−	+	+	−	I	2	5
5593	BF	+	+	+	+	++	I	2	5
5595	BF	+	+	+	+	++	II	17	6
5794	CSF	+	+	−	ND	−	III	532	1
6076	CSF	+	+	−	ND	−	III	88	1
6152	CSF	+	+	−	ND	−	III	294	1
6381	Eye	+	+	−	ND	+	II	5	6
6439	CSF	+	+	−	ND	+	II	5	6
6802	TC	+	+	−	ND	−	III	73	1
6836	BF	+	+	+	−	−	I	2	5
7022	TC	+	+	−	ND	+	II	5	6
7109	BF	+	+	−	ND	+	III	218	1
7235	TC	−	ND	−	ND	+	I	256	1
7237	CSF	−	ND	−	ND	−	I	534	4
7327	CSF	+	+	−	ND	+	II	5	6
7463	TC	+	+	−	ND	+	II	5	6
7486	TC	+	+	−	ND	−	I	153	6
7613	CSF	+	+	+	+	++	I	166	1
7736	CSF	−	ND	−	ND	−	II	ND	ND
8006	CSF	−	ND	−	ND	−	II	ND	ND
8099	BF	+	+	−	ND	−	III	73	1
8111	BF	+	+	−	ND	−	III	73	1
8364	TC	+	+	−	ND	−	III	88	1
8500	TC	+	+	−	ND	−	II	5	6
8571	TC	+	+	−	ND	−	II	5	6
8890	TC	+	+	+	+	+++	I	16	1
8899	CSF	−	ND	−	ND	−	I	537	4
9958	BF	+	+	+	+	+	I	16	1
9973	CSF	−	ND	−	ND	−	I	ND	ND
9987	Eye	+	+	−	ND	+	II	5	6
10017	CSF	+	+	−	ND	+	II	5	6
10101	BF	−	ND	−	ND	−	I	89	1
10340	CSF	+	+	−	ND	+	I	487	6
10413	BF	+	+	+	−	−	I	2	5
10532	BF	+	+	+	+	+	I	2	5
10596	SF	+	+	−	ND	−	II	152	1
10734	SF	−	ND	−	ND	−	II	7	6
O15	Expl	+	+	−	ND	+	II	5	6
O16	Expl	+	+	−	ND	−	I	57	6
O50	Expl	+	+	−	ND	++	II	5	6
O73	Expl	−	ND	+	+	+++	I	9	1
O101	Expl	+	+	−	ND	−	II	5	6
O135	Expl	−	ND	−	ND	+	I	ND	ND
O136	Expl	+	+	−	ND	+	II	5	6

a Blood isolates were from patients with concomitant positive catheter tip culture results. ID, identifier; BF, body fluid other than blood; CSF, cerebral spinal fluid; TC, tissue culture from site of prosthetic joint removal; SF, synovial fluid; Expl, explanted hardware (knee or hip).

b Biofilm formation compared to S. epidermidis 1457: −, >30%; +, 30% to 59%; ++, 60% to 84%; +++, ≥85%.

c ST, sequence type determined by MLST.

d GC, genetic cluster based on BAPS of MLST.

e ND, not determined. In columns 3 to 6, + indicates presence and − indicates absence.

### Production of matrix components.

It was next determined if the presence of *aap* or *icaADBC* correlated with synthesis of Aap or PIA, respectively. Accordingly, we performed Western blotting using serum raised against Aap, as well as PIA immunoblotting, to identify matrix components produced by the isolates. Aap was detected by Western blotting in 74 of the 80 *aap*-positive isolates (92.5%), indicating high concordance between the presence of the gene and protein production (Tables 1 and 2). In contrast, PIA was detected in only 67% (24/36) of the *ica*-positive isolates (Tables 1 and 2).

### Biofilm phenotypes differ between shear groups.

A static microtiter plate assay was used to test the ability of isolates to produce biofilm *in vitro*. Due to the large number of isolates tested and the inherent variability of the assay, biofilm formation was normalized by comparing it to that of robust biofilm-forming isolate 1457. Biofilm formation was stratified into the following 4 groups based on comparison to strain 1457: negative (–; <30%), slight (+; 30 to 59%), moderate (++, 60 to 84%), and strong (+++; ≥85%). Overall, 34.3% of the isolates were negative for biofilm formation in the static assay, 45.7% showed slight biofilm formation, 8.6% showed moderate biofilm formation, and 10.4% showed strong biofilm formation. Among the isolates in the high-shear group, 17.5% were biofilm negative, 57.9% showed slight biofilm formation, 10.4% showed moderate biofilm formation, and 17.5% showed strong biofilm formation. In the low-shear group, 54.2% of the isolates were biofilm negative, 31.3% showed slight biofilm formation, 10.4% showed moderate biofilm formation, and 4.2% showed strong biofilm formation. Within the high-shear group, 47 strains (82.5%) were biofilm positive (slight/moderate/strong), while less than half of the low-shear isolates (22 of 48, 45.8%) produced biofilm (*P* < 0.05). Additionally, 14.6% of the low-shear isolates and 24.6% of the high-shear isolates were either moderate or strong biofilm producers (*P* < 0.05). Similarly, 4.2% of the low-shear and 17.5% of the high-shear isolates were strong biofilm producers (*P* < 0.05). In summation, there was a significant association between biofilm formation and infection site; those isolates obtained from a proposed high-shear environment produced significantly more biofilm than those isolated from a low-shear infection site. While the exact relationship between biofilm formation in the static assay and *in vivo* relevance is not clear, our results and those of others (45) have demonstrated that the isolates identified as the strongest biofilm formers (+++) carry the *icaADBC* operon and produce large amounts of PIA without induction under environmental conditions such as the presence of excess salt or ethanol. Indeed, PIA was detected in all 12 of the isolates that were very strong biofilm producers (Tables 1 and 2). Taken together, these data support the idea of a relationship between constitutive PIA expression and the ability to colonize catheters in a high-shear environment.

### *icaADBC* transcription is induced upon exposure to shear stress.

**FIG 2:**
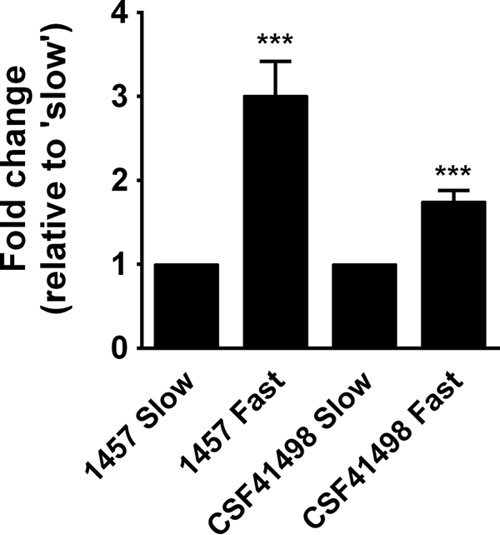
*icaA* expression increases as shear flow increases. Strain 1457 or CSF41498 biofilms were grown in convertible flow cells at either high (“slow”) or low (“fast”) rates of flow for 24 h at 37°C. Biofilms were harvested, and RNA was purified for qRT-PCR analysis. *icaA* expression was higher with “fast” flow than with “slow” flow. ***, *P* < 0.05.

Our results suggest that the presence of *icaADBC* and enhanced PIA synthesis may be advantageous in environments that contain high shear stress. Indeed, previous data suggest that, in contrast to wild-type (WT) strain 1457, the 1457 Δ*icaADBC* strain is unable to form a biofilm in a high-shear flow cell (32). Therefore, to determine whether increased shear induces *icaADBC* expression, two *S. epidermidis* clinical isolates, 1457 (high constitutive PIA production) and CSF41498 (inducible PIA production), were grown in convertible flow cells at either low (0.25 ml/min) or high (2 ml/min) shear flow rates. When grown under high-shear flow conditions, *icaA* transcription was increased 3.0087-fold in 1457 (*P* < 0.05) and 1.7471-fold in CSF41498 (*P* < 0.05) compared to growth under low-shear flow conditions (Fig. 2). These data demonstrate that shear flow impacts biofilm formation via induction of genes responsible for PIA synthesis. In addition, this observation may provide a mechanistic explanation for apparent discrepancies between the observation of biofilm formation *in vivo* and the apparent lack of biofilm formation in static assays and other *in vitro* assays.

### Selection of biofilm-enriched variants.

Within the high-shear group, there were 32 *ica*-negative strains, of which 65.6% (*n* = 21) were slight biofilm producers (+) and 3.1% (*n* = 1) were moderate biofilm producers (++). In fact, it is well documented that many clinical isolates that cause infection lack the *icaADBC* operon (30, 34, 37, 47, 54, 68–70). Therefore, we asked if it would be possible to enrich for variants with increased biofilm formation among the members of a population that are *ica* negative and, therefore, generally poor biofilm producers. To explore this possibility, tissue culture flasks were inoculated with the PIA-deficient, slight-biofilm-forming 1457 Δ*icaADBC* strain (referred to here as strain 1457 Δ*ica*) (71). Through a series of wash and incubation steps, mutants able to adhere to the flask surface were selected, and after isolation of individual colonies, biofilm-positive variants were identified using microtiter plate assays. Of the 209 isolates tested from 3 separate enrichment experiments, 91 formed significantly more biofilm than the parent strain (1457 Δ*ica*) using a static microtiter assay.

**FIG 3:**
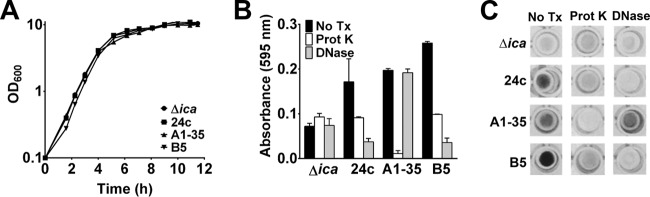
Enriched-variant dispersal. (A) Growth analysis in TSB of the 1457Δ*ica*, 24c, B5, and A1-35 strains. (B) The 24-h biofilms were treated with enzymes for 2 h and then processed for biofilm formation analysis. (C) Representative images of biofilms following crystal violet staining. Prot K, proteinase K; No Tx, no treatment.

Three of the strain 1457 Δ*ica* variants, 24c, A1-35, and B5, were selected for further analysis because they showed various degrees of biofilm enhancement, their biofilm phenotypes were stable, and they were isolated from 3 separate enrichment procedures. First, to confirm that increased biofilm formation was not merely a consequence of growth enhancement, growth experiments were performed on the 3 enriched isolates and the 1457 Δ*ica* strain. The results confirmed that the enriched isolate growth rate or yield did not differ drastically from that seen with the parent strain (1457 Δ*ica*) (Fig. 3A), suggesting the observed biofilm phenotypes were specific to biofilm formation and thus warranted further investigation. To assess the composition of the biofilm matrices, 24-h biofilms were treated with either proteinase K or DNase I (Fig. 3B and C). The parent strain, 1457 Δ*ica*, was not affected by treatment with proteinase K or DNase I. DNase I also had no effect on A1-35; however, proteinase K ablated the preformed biofilm. Biofilms of 24c and B5 were decreased to the levels seen with the parent strain following treatment with proteinase K, while DNase I treatment caused the greatest dispersal. These data suggest that the matrix of A1-35 is comprised primarily of protein, while both 24c biofilms and B5 biofilms are eDNA and protein dependent.

**FIG 4:**
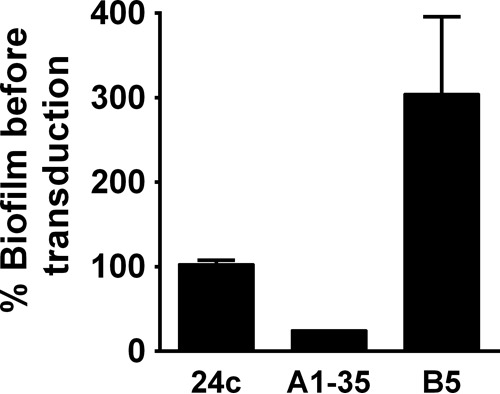
Deletion of *aap* negates biofilm enhancement in A1-35. Levels of biofilm formation in enriched variants before and after allelic replacement (*aap*::*tetM*) were compared to examine the contribution of Aap to the biofilm phenotype. Biofilm formation in the static microtiter assay was assessed by absorbance at 595 nm following crystal violet staining.

To determine if Aap was involved in the enriched-variant phenotypes, lysate was made from strain 1457 Δ*aap* (72) and transduced into strains 24c, A1-35, and B5. Deletion of *aap* did not impair biofilm formation in 24c or B5; however, strain A1-35 Δ*aap* biofilm levels were reduced to the level seen with the parent strain, indicating that the enhanced biofilm formation in variant A1-35 was Aap dependent (Fig. 4). This suggests that A1-35 biofilm enrichment is due to alteration of Aap or to factors controlling its expression and/or processing. Although biofilms produced by variants 24c and B5 were disrupted following proteinase K treatment, the results of the transduction experiment indicated that Aap is not responsible for the increased biofilm production in these strains, suggesting that alternative proteinaceous factors are involved.

### Growth of enriched isolates under fluid shear conditions.

**FIG 5:**
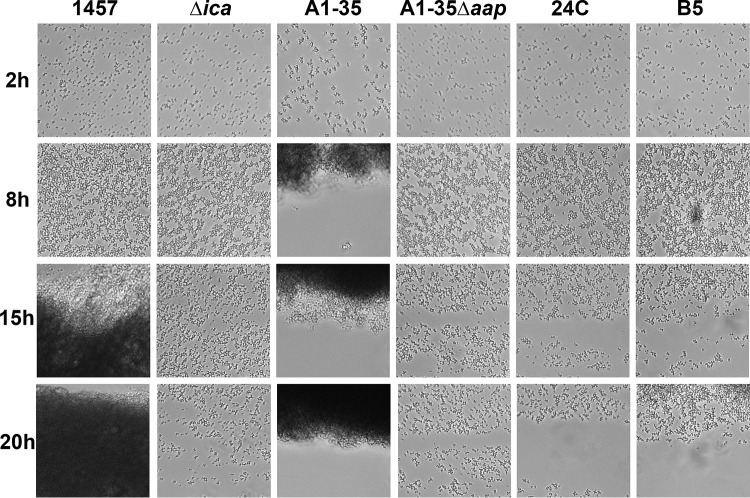
1457 and A1-35 form robust biofilms under shear flow conditions. At 2 h after inoculation, A1-35 cells were clustered and had a jagged appearance, while the cells of all other strains were spread more diffusely across the surface. After 8 h, the phenotypes of the 1457, Δ*ica*, 24c, B5, and A1-35 Δ*aap* strains were still comparable, while increased cell-cell interactions were apparent in strain A1-35. By 15 h, the effect of PIA production was apparent in strain 1457. A larger cell aggregate had developed in A1-35, while Al-35 Δ*aap*, 24c, and B5 cells were less able to withstand fluid movement, resulting in bare streaks. The enhanced intercellular interactions of strain A1-35 gave the cells an appearance of “snowflaking,” or spreading outward. At 20 h after inoculation, fluid flow through the channel containing strain 1457 was blocked by PIA-mediated biofilm and A1-35 cells had formed a compact biofilm extending from the edge of the channel.

The ability of the variants to grow under shear conditions (10 dyne/cm^2^) was assessed using a BioFlux microfluidic plate system and automated image acquisition. At 2 h, A1-35 cells were found in clusters with a jagged appearance, while cells of all other strains were located more diffusely across the plate surfaces (Fig. 5). After 8 h, the phenotypes of strains 1457, 1457 Δ*ica*, 24c, B5, and A1-35 Δ*aap* were still comparable, while increased cell-cell interactions were apparent in strain A1-35. By 15 h, the effect of PIA production was apparent in strain 1457. Cell aggregation was further increased in strain A1-35, while cells of strains A1-35 Δ*aap*, 24c, and B5 were less able to withstand fluid shear, as evidenced by the presence of bare streaks across the viewing windows. As a consequence of the enhanced intercellular interactions of A1-35, daughter cells remained closely associated for multiple generations. In fact, in time-lapse images, cell clusters appeared to “snowflake,” spreading outward in a manner reminiscent of ice crystal formation. At 20 h after inoculation, fluid flow through the microfluidic channel containing strain 1457 was completely blocked by PIA-mediated biofilm. Variant A1-35 had formed a compact biofilm extending from the edge of the channel, and, to a lesser degree, 24c and B5 cells had started to accumulate along the channel edges. Of note, deletion of *aap* in strain A1-35 restored the phenotype to that of the parent strain, 1457 Δ*ica*. Interestingly, cell accumulation and biofilm formation were most evident at the edges of channels, where fluid shear is decreased; however, the intercellular interactions were not strong enough to allow clustering in the center of the channels. This is in agreement with previous findings indicating that protein-mediated biofilms were not as robust as those containing PIA (32). The flow cell results provide further evidence that the increased biofilm formation of strain A1-35 is Aap dependent, possibly due to an increase in Aap-mediated cell-cell interactions.

### Sequence analysis of biofilm-enriched variants.

Due to abrogation of biofilm formation in A1-35 following introduction of the *aap* mutation, we hypothesized that the isolate contained changes in *aap* or factors regulating its expression or processing. DNA sequencing analysis identified the point mutation G1363T in *aap*, thereby coding for cysteine rather than glycine at residue 455, slightly less than halfway into the L-type lectin domain. Based on phenotypic analysis of variant A1-35, it appears that the substitution increases Aap-dependent interactions between cells. Genetic analysis of variant 24c revealed a single nucleotide change from guanine to adenine, causing the amino acid change L45F in GlcNAc 2-epimerase. GlcNAc 2-epimerase is a highly conserved enzyme (73, 74) responsible for the conversion of GlcNAc to UDP-*N*-acetylmannosamine (ManNAc) (74–76). Finally, the third biofilm-enriched variant, B5, had 2-bp substitutions, resulting in the amino acid changes D498Y and Y497F within the amidase domain of the autolysin AtlE (77).

## DISCUSSION

We began this study by assessing population-based questions associated with disease-causing *S. epidermidis* clinical isolates collected from high-shear and low-shear environments. Overall, we found no biologically significant population-based differences between these groups of isolates in studies employing multilocus sequence typing (MLST) or determination of the *agr* group. However, several observations deserve notice. Within our isolate population, the most frequent ST was ST5 (*n* = 26), followed by ST2 (*n* = 14) and ST7 (*n* = 5). ST5 contained almost exclusively *aap*-positive strains (25 of 26), of which 24 produced Aap. Interestingly, none of the strains carried the *icaADBC* locus. Twenty-five isolates were *agr* type II, while 1 was type I. High-shear and low-shear environments were equally represented (13 of each), and both shear groups primarily contained isolates that were minimal biofilm producers (+), 1 isolate that was a moderate biofilm producer (++), and 3 strains that did not make biofilm. All ST2 isolates were positive for *aap*, and, in agreement with previous findings, 13/14 were *icaADBC* positive (29, 52, 60). All ST2 isolates were *agr* type I. There were 9 high-shear isolates, among which 7 were minimal biofilm producers (+), 1 was a moderate biofilm producer (++), and 1 was biofilm negative, as well as 5 low-shear isolates, 1 each of minimal (+) and moderate (++) biofilm producers and 3 that were biofilm negative. Juárez-Verdayes et al. (78) observed an association between biofilm composition and strain lineage, as 50% of ST2 biofilms were composed of protein, or of a combination of protein and eDNA, and such biofilms are known to be less robust than those containing primarily PIA. Our findings do not contradict this, as all the ST2 isolates were *aap* positive, with 13 of 14 producing Aap. Among the 5 ST7 strains, 1 was low shear and had characteristics different from those of the high-shear strains. The low-shear isolate was *aap*, *ica*, and biofilm negative and *agr* type II positive. All high-shear strains produced PIA and were strong biofilm producers (+++), 3 of 4 contained *aap*, and 3 were *agr* type I whereas 1 was *agr* type II.

Regarding genetic cluster analysis, consistent with previous reports, we found that 93% of isolates tested were GC1 or GC5 or GC6 (61, 79). Although trends were noted with respect to specific genetic clusters and high- or low-shear isolates (e.g., for GC1 and high shear, *n* = 8; for GC1 and low shear, *n* = 16), none of these associations were statistically significant. However, two previous reports of *S. epidermidis* isolates from New York and Illinois found an association between GC5 and the presence of *icaADBC* (61, 79). Our data are consistent with these observations in that 16/17 (94%) of the GC5 isolates in our study carried *icaADBC*, whereas only 33% of the isolates overall carried this locus*.*

A total of 76% of the clinical isolates carried *aap*, and nearly all (93%) of those produced Aap as detected by Western blotting. Aap and its *S. aureus* homologue, SasG, have both been shown to facilitate binding to host epithelial cells (80–82), suggesting a function during colonization. This hypothesis is supported by the high prevalence of *aap*-positive strains detected in our study. To date, no direct regulators of *aap* have been identified. In light of our findings, it seems probable that production of Aap in *aap*-positive isolates is typical and that deviations from this are the exception.

In contrast to our observations regarding Aap, PIA was detected in only two-thirds (24/36) of the *ica*-positive isolates, which is in agreement with previous findings (83). Among the *S. epidermidis* isolates, 43.9% of those obtained from a high-shear environment, in contrast to 22.9% of those obtained from a low-shear environment, carried the *icaADBC* operon. The synthesis of PIA is an energy-intensive process involving multiple proteins and diverts glycolytic substrate away from cell wall synthesis and from pyruvate and subsequent central metabolism. Therefore, it is not surprising that transcription of *icaADBC* is regulated by multiple factors, including those that are linked to central metabolism (19, 84). Although the great majority of *S. aureus* strains carry the *icaADBC* operon, it is rarely synthesized *in vitro* without induction (22). Furthermore, as documented in these studies, many *S. epidermidis* isolates that carry the *icaADBC* operon do not produce detectable PIA or strongly produce (+++) biofilm. Nevertheless, 10 of the 12 isolates that formed biofilm most strongly (+++) from this study were from high-shear environments and all 12 produced detectable PIA. Further flow cell studies demonstrated that increased flow induced *icaADBC* transcription in two separate strains of *S. epidermidis*, 1457 and CSF41498. Note that 1457 produces abundant PIA *in vitro*, whereas CSF41498 requires induction by salt or ethanol (33, 34). Collectively, these data suggest that carriage and subsequent induction of the *icaADBC* operon are advantageous under conditions of high shear stress such as those found within the lumen of a catheter. Therefore, we propose that one main function of PIA is to protect the biofilm structure and ensure structural integrity under conditions of high shear stress. Furthermore, ill-defined mutations that enhance *icaADBC* transcription and subsequent PIA synthesis, as described for strain 1457 and for 12 strains in this report, may also enhance the ability of *S. epidermidis* to colonize and withstand certain shear stress conditions. The factors that mediate phenotypic differences in PIA synthesis between 1457- and CSF41498-like strains are under active investigation.

It is very clear that staphylococcal biofilm accumulation can be mediated by multiple molecules, including polysaccharide, protein, or eDNA. These observations suggest that biofilm accumulation molecules are redundant and that certain accumulation strategies may be selected within certain niches. Highlighting this point, it has been previously demonstrated that the same strain of *S. epidermidis* that produced biofilm dependent upon different matrix molecules was isolated multiple times from a single patient (35). Regarding PIA synthesis, data from the present study suggest that *icaADBC* carriage and subsequent induction may be advantageous in niches of high shear stress. However, data exist suggesting that PIA expression is deleterious to colonization of the skin (85). Indeed, although it is unclear how *S. epidermidis* may acquire *icaADBC*, Ziebuhr and colleagues and our group independently determined that *icaADBC* is lost within a population due to chromosomal deletion via recombination of insertion sequence elements (86, 87). Therefore, as it is clear that there are redundancies in biofilm accumulation molecules and variations in the amount of biofilm produced within any given strain, experiments were designed to identify loci responsible for enhancing biofilm formation in a PIA-deficient strain, the 1457 Δ*ica* mutant*.* The genomes were sequenced from three 1457 Δ*ica* mutants able to produce significantly more biofilm than the wild-type strain following an enrichment assay. Surprisingly, single nucleotide polymorphism (SNP) analysis identified only one mutation in strains A1-35 (*aap*) and 24c (GlcNac 2 epimerase) and two mutations within the same gene in mutant B5 (AtlE). Although it is unclear how any of these mutations function to increase biofilm formation of the 1457 Δ*ica* mutant, it is important that multiple mechanisms can be selected to enhance cell aggregation in *S. epidermidis*. First, the G1363T mutation in *aap* of A1-35 is found within the lectin domain, which may function in initial adherence to plastic or to other proteins/glycoproteins (72, 82). However, it is difficult to envision how this mutation could facilitate increased cellular interactions as the Zn^2+^-dependent homophilic bonds occur between the G5-E domains within the B region but not within the lectin binding domain (88, 89). Second, growth inhibition resulting from inactivation of GlcNAc 2-epimerase has been documented in many Gram-positive species, including *S. aureus* and *S. epidermidis* (74). Proper regulation of cell wall turnover is critical for cell growth and viability; thus, it seems reasonable to conclude that increased activity of GlcNAc 2-epimerase would also be detrimental. We hypothesize that the point mutation increases the activity of GlcNAc 2-epimerase, thereby increasing cell wall turnover and eDNA release following cell lysis. Indeed, 24c biofilm was almost completely dispersed following DNase I treatment. Finally, we propose that the mutations in the amidase domain of AtlE in B5 may enhance the activity of the enzyme, increasing cell lysis and eDNA release. We are currently investigating whether the observed increased biofilm formation of B5 containing an *aap* mutation (Fig. 4) is relevant in other relevant biological assays, including animal models of infection. While the events leading to release of eDNA differ between 24c and B5, the two biofilms were dispersed by DNase I and had similar phenotypes in the BioFlux assay, supporting the idea of a shared enhanced biofilm formation mechanism. However, it is important that there was no difference between 24c and B5 with respect to growth yield following 12 h of growth in comparison to the WT results (Fig. 3A), suggesting that if our supposition is correct, increased cell lysis may occur at different stages of growth (biofilm or enhanced stationary phase) instead of occurring during exponential growth in broth.

## MATERIALS AND METHODS

### Bacterial culture conditions.

The *S. epidermidis* clinical isolates used in this study are listed in Tables 1 and 2. Other *S. epidermidis* strains used included 1457 (33) and CSF41498 (34). Staphylococcal strains were cultured in tryptic soy broth (TSB; Becton, Dickinson) or tryptic soy agar (TSA). When appropriate, antibiotics were added to media at a concentration of 10 µg/ml. Unless otherwise stated, cultures were grown aerobically (1:10 media-to-flask ratio, 250 rpm) at 37°C. If not specified, all chemicals and reagents were purchased from Sigma (St. Louis, MO).

### Phage propagation and transduction.

The 1457 *aap* mutation (72) was transferred to biofilm-enriched variants using transducing phage 71 (90) as previously described (91).

### Pulsed-field gel electrophoresis (PFGE).

Pulsed-field gel electrophoresis was performed using previously described methodology (92). Briefly, high-molecular-weight DNA encased within agarose plugs was made by combining bacterial suspensions of an optical density at 600 nm (OD_600_) of 1.0, lysostaphin (25 µg), and 1% pulsed-field-certified agarose (Bio-Rad, Richmond, VA). Following a 4-h incubation in EC buffer (6 mM Tris hydrochloride, 1 M NaCl, 100 mM EDTA, 0.5% Brij 58, 0.2% deoxycholate, 0.5% sodium lauroyl sarcosine [pH 7.5]) at 37°C, plugs were transferred to ESP buffer (0.5 M EDTA [pH 9.0 to 9.5], 1% sodium lauroyl sarcosine) containing 20 µg proteinase K and incubated overnight at 54°C. Finally, plugs were washed and stored in Tris-EDTA (TE) at 4°C until use. Plugs were digested with SmaI (Thermo Scientific) before incorporation into a 1% agarose gel. The gel was run in 0.5× Tris-borate-EDTA (TBE) in an electrophoresis cell using a CHEF-DR III system (Bio-Rad) and the following parameters: 6 V/cm; initial pulse time, 1 s; final pulse time, 30 s for 22 h at 14°C. Gels were visualized by UV exposure following ethidium bromide staining.

### Static biofilm assay.

Microtiter plate assays were performed as described previously (72) based on the procedure of Christensen et al. with modifications (43, 93). Overnight planktonic cultures were diluted 1:100 in fresh TSB. Each well of a 96-well flat-bottom microtiter plate was inoculated with 200 µl, with samples run in triplicate. Plates were incubated statically at 37°C for 24 h. The supernatant was removed, and wells were washed three times with phosphate-buffered saline (PBS). Cells were fixed by drying plates at 45°C for 1 h. Adherent cells were stained with 0.4% crystal violet for 15 min, and excess stain was removed by washes in distilled water. Biofilm formation was quantified by measuring the absorbance at 595 nm using a Wallac 1420 multilabel counter (PerkinElmer, Waltham, MA). Results presented are from at least 3 independent experiments.

### Biofilm dispersal.

Twenty-four-hour biofilms, inoculated and grown as described above, were treated with enzymes for 2 h at 37°C as follows. Medium was removed from wells before the addition of 100 µl of 40 µg/ml DispersinB–PBS (MTA-Kane Biotech Inc., Winnipeg, Manitoba, Canada) (94), 100 µg/ml proteinase K (Invitrogen, Carlsbad, CA)–20 mM Tris-HCl (pH 7.5)–100 mM NaCl, or the corresponding buffers. DNase I requires Mg^2+^ for maximal activity, so for this treatment, 100 µl of TSB (which contains Mg^2+^) was removed, leaving 100 µl in the wells, to which 3 µl of 10 U/µl DNase I (Roche, Indianapolis, IN) was directly added. Cells in TSB with no enzyme added served as the control for DNase I. After treatment, biofilms were processed and analyzed as described for the static biofilm assay.

### Biofilm harvest and protein isolation.

Twenty-four-hour TSA plates were used for inoculation of sterile saline solution to reach an absorbance of 0.06 (Microscan turbidity meter; Siemens). Volumes of 50 µl of the bacterial suspensions were added to 5 ml TSB in 6-well polystyrene cell culture plates (Corning Incorporated, Corning, NY), and the plates were incubated statically at 37°C for 24 h. Medium was removed and added to 5-ml tubes, and 2 ml of PBS was added to each well. Spent medium was centrifuged twice to remove bacterial cells. Extracellular fractions were then concentrated using 100-molecular-weight-cutoff (MWCO) columns (Millipore, Billerica, MA) according to the manufacturer’s instructions. Cells were removed from wells using cell scrapers (Nalge Nunc, Rochester, NY) and pipetted into 2-ml tubes. Protein isolation procedures were performed based on previously published methods (80, 81, 95) with modifications. Cells were pelleted and washed in PBS twice and then resuspended in 1-ml lysis buffer (50 mM Tris-HCl, 20 mM MgCl_2_ [pH 7.5] with 30% [wt/vol] raffinose, containing 1 mM EDTA, 20 U DNase I [Roche], 4-mM phenylmethylsulfonyl fluoride, 1 mM *N*-ethylmaleimide, 25 mM aminocaproic acid, lysostaphin [100 µg/ml; AMBI Products, Lawrence, NY], and lysozyme [100 µg/ml]) and incubated at 37°C for 30 min. Cell walls (CW) were separated from protoplasts by centrifugation at 6,000 × *g* for 20 min. The supernatant (cell wall fraction) was removed and concentrated using 100-MWCO columns (Millipore) according to the manufacturer’s instructions. Protein concentrations were determined using an ND-1000 spectrophotometer (NanoDrop, Wilmington, DE) prior to storage of concentrated samples at −20°C.

### SDS-PAGE and Western blotting.

For each sample, 2.5 µl Laemmli buffer (96) containing β-mercaptoethanol was added to approximately 50 µg total protein and the final volume was adjusted to 15 µl with Tris-buffered saline (TBS) as needed. Samples were boiled for 10 min and then immediately placed on ice. The entire volume prepared for each sample was loaded per well, and 20 µl of Spectra multicolor high-range protein ladder (Fermentas, Pittsburgh, PA) was loaded for size determination. Proteins were separated on 5% SDS-PAGE mini gels (1.5-mm thickness) by electrophoresis at 90 V for 15 min and then at 40 V for approximately 12 h at 4°C. Gels were stained in Bio-Safe Coomassie G-250 stain (Bio-Rad) according to the manufacturer’s instructions. Proteins were transferred to polyvinylidene difluoride (PVDF) membrane by wet transfer at 30 mA for 45 min. Membranes were blocked with 5% nonfat milk–TBS–Tween. Aap B domain antiserum (30) was diluted 1:100,000 in 5% nonfat milk. Alkaline phosphatase (AP)-conjugated mouse anti-rabbit IgG (Jackson ImmunoResearch, West Grove, PA) was diluted 1:25,000 in 5% nonfat milk. Blots were washed after incubation with primary and secondary antisera in either TBS-Tween or TBS. Chemiluminescence was visualized following application of ECF substrate (GE Healthcare Life Sciences, Piscataway, NJ) using a Typhoon FLA 7000 laser scanner (GE Healthcare Life Sciences).

### Flow cell biofilms.

Stovall convertible flow cells were inoculated with approximately 10^5^ cells of wild-type 1457 or CSF41498. Cells were allowed to adhere for 30 min at 37°C prior to initiation of medium flow. Biofilms were grown at 37°C in 100% TSB under conditions of constant flow for 24 h at a rate of either 0.25 ml/min (“slow”) or 2 ml/min (“fast”). Biofilms were harvested by removing TSB from the flow cell chamber and resuspending biofilm cells in 900 µl of RLT buffer (Qiagen)–1% β-mercaptoethanol. Cells were disrupted using a FastPrep FP120 device (Thermo Scientific). RNA isolation and purification were performed using an RNeasy minikit (Qiagen, Venlo, Limburg, The Netherlands). cDNA was generated with a QuantiTect reverse transcription kit (Qiagen, Venlo, Limburg, The Netherlands) using ~500 ng of RNA per sample. cDNA was diluted 1:20, and quantitative real-time PCR (qRT-PCR) was performed using a LightCycler 480 SYBR green I Master kit (Roche), *gyrB* primers as the control, and *icaA*-specific primers for detection of *ica* expression. qRT-PCR was performed on a LightCycler 480 II instrument (Roche). Transcript levels are normalized to *gyrB* levels, and *icaA* levels from “fast” samples are reported as fold change compared to “slow” samples. Data are averages from three biological replicates.

### Clinical isolate selection.

Isolates were obtained from patient samples collected between 2007 and 2012 at Nebraska Medicine hospitals, with only 1 isolate per patient included. Isolates were identified as *S. epidermidis* using a combination of standard phenotypic methods (Microscan, Beckman Coulter) and PCR primers that amplify a unique chromosomal segment of *S. epidermidis* (primers 2332 and 2333; Table 3) (97). A total of 105 isolates were included in the study, consisting of 57 from high-shear and 48 from low-shear environments. In the high-shear group, isolates were from confirmed positive blood cultures (at least 2 of 3 positive blood bottles) from patients with concomitant positive catheter tip cultures. PFGE was utilized to confirm that the blood isolate and catheter tip isolate were indistinguishable. Low-shear isolates were from body fluid (*n* = 14), cerebrospinal fluid (*n* = 13), eye (*n* = 2), tissue (*n* = 5), and samples obtained during or immediately following removal of prosthetic devices (*n* = 14).

**TABLE 3:** Primers used in this study

Primer	Sequence	Amplified gene	Reference or source
8	GGTCGTGACATATGAAACC	*icaB*	This study
9	CGAATCCGTCCCATTCC	*icaB*	This study
1838	TCTAATGGCCAAGATTTCACG	*ureD*	102
1839	TGAAACTTTGGTTTACATCTGGA	*ureD*	102
1840	CGCGAAGCCCCTACAAGAAATGACCTAGC	*aap*	This study
1841	CGCGCTGTTGTTGTACCAGGTGGCTGTCC	*aap*	This study
2301	CTCGAAGCGGTTCGTAAAAG	*gyrB*	This study
2302	TACCACGGCCATTGTCAGTA	*gyrB*	This study
A1 (2160)	GCTGCAACCAAGAAACAACC	*agr* types I, II, III	66
A2 (2161)	CGTGTATTCATAATATGCTTCGATT	*agr* types I, II, III	66
B1 (2162)	TATGCAAGCCAAGCACTTGT	*agr* types II, III	66
B2 (2163)	GTGCGAAAGCCGATAACAT	*agr* types II, III	66
C1 (2164)	CCTTGGCTAGTACTACACCTTC	*agr* type II	66
C2 (2165)	GTGCTTGGCTTGCATAAACA	*agr* type II	66
2332	ATCAAAAAGTTGGCGAACCTTTTCA	*S. epidermidis*-specific sequence	97
2333	CAAAAGAGCGTGGAGAAAAGTATCA	*S. epidermidis*-specific sequence	97
2336	GCTACAATGAAAGTGAAAC	*icaA*	This study
2337	GGCACTAACATCCAGCATAG	*icaA*	This study
2372	TGTGATGAGCACGCTACCGTTAG	*arcC*	103
2373	TCCAAGTAAACCCATCGGTCTG	*arcC*	103
2374	CATTGGATTACCTCTTTGTTCAGC	*aroE*	103
2375	CAAGCGAAATCTGTTGGGG	*aroE*	103
2376	CAGCCAATTCTTTTATGACTTTT	*gtr*	103
2377	GTGATTAAAGGTATTGATTTGAAT	*gtr*	103
2378	GATATAAGAATAAGGGTTGTGAA	*mutS*	103
2379	GTAATCGTCTCAGTTATCATGTT	*mutS*	103
2380	GTTACTAATACTTTTGCTGTGTTT	*pyrR*	103
2381	GTAGAATGTAAAGAGACTAAAATGAA	*pyrR*	103
2382	ATCCAATTAGACGCTTTAGTAAC	*tpi*	103
2383	TTAATGATGCGCCACCTACA	*tpi*	103
2384	CACGCATAGTATTAGCTGAAG	*yqiL*	103
2385	CTAATGCCTTCATCTTGAGAAATAA	*yqiL*	103

### Isolation of chromosomal DNA.

Chromosomal DNA was isolated using a blood and tissue kit (Qiagen) with modifications. Volumes of 2 ml of overnight cultures were pelleted and resuspended in 500 µl TE buffer and then transferred to tubes containing 0.1-mm-diameter glass beads. Cells were lysed by 2 cycles of manual disruption for 20 s per cycle at speed 6.0 in a Thermo Savant FastPrep FP120 cell disruptor (MP Biomedicals, Santa Ana, CA) with a period of 5 min on ice between cycles. Cell lysates were centrifuged at 14,000 × *g* in bead beater tubes for 5 min. The supernatant (275 µl) was transferred to a new 1.5-ml tube, and 38.5 µl proteinase K and 305 µl buffer AL (Qiagen) were added. The mixtures were incubated at 60°C for 30 min, after which the isolation procedure was completed according to the manufacturer’s instructions. Chromosomal DNA was eluted in sterile H_2_O and stored at −20°C until use.

### PCR screening.

PCR was performed using Midas Mix with Taq DNA polymerase I (Monserate Biotechnology Group) and 10 µM primers. Primers 1840/1841, 2336/2337, and 8/9 were used to amplify *aap* (347 bp; annealing temperature of 50°C with 30-s extension), *icaA* (597 bp; annealing temperature of 50°C with 30-s extension), and *icaB* (870 bp; annealing temperature of 53°C with 50-s extension), respectively (Table 3). The presence of *icaADBC* was confirmed using an *icaA* dot blot. Briefly, 3 µl denaturing solution (4 M NaOH and 100 mM Na_2_-EDTA) was added to 27 µl water containing 750 ng chromosomal DNA and the reaction mixture was incubated at room temperature for 10 min. Denatured sample (5 µl) was spotted onto a positively charged nylon membrane (Roche Diagnostics) and allowed to dry. DNA was fixed using a Stratalinker UV 1800 Crosslinker (Stratagene), and the blot was hybridized with a digoxigenin (Roche, Indianapolis, IN)-labeled *icaA* DNA probe (amplified using primers 2336/2337; Table 3) and subsequently developed using the recommendations of the manufacturers.

### *agr* typing.

Isolates were typed using the methodology developed by Li et al. (66). The reaction annealing temperature was 55°C, with a 1-min extension repeated for 25 cycles. Three groups of primers (A, B, and C) with different specificities for sequences within the variable region of *agrD* were used to amplify genomic DNA. The primers were designed such that the A primers (2160/2161) amplified a 1,022-bp region common to all 3 *agr* types, the B primers (2162/2163) amplified 453 bp present only in types II and III, and the C primers (2164/2165) amplified 615 bp specific to type II. The PCR typing methodology was confirmed by sequencing representative isolates from each of the 3 *agr* types.

### PIA immunoblots.

Bacterial cultures were adjusted to an OD_600_ value of 5, subsequently pelleted by centrifugation at 6,000 × *g* for 5 min, and then resuspended in 500 µl of 0.5 M EDTA and boiled for 5 min. Cells were pelleted, and 40 µl of supernatant was transferred to new tubes. A 10-µl volume of proteinase K (20 mg/ml) was added to tubes prior to incubation at 37°C for 1 h. Samples were boiled for 5 min and then stored at −20°C until use. Immobilon-P PVDF membrane (Millipore) was primed in methanol and then transferred to TBS. The membrane was placed in a Bio-Dot apparatus (Bio-Rad) and dried briefly by vacuum. A 300-µl volume of TBS was added to each well and pulled through the membrane by vacuum. A 100-µl volume of sample was applied to each well, liquid was removed by vacuum, and membranes were dried to fix samples. To detect PIA, membranes were rehydrated in TBS and then blocked in 5% nonfat milk–Tris-buffered saline with Tween 20 (TBST) at room temperature for 2 to 4 h. Primary antibody (kind gift of Jim O’Gara, National University of Ireland, Galway, Ireland), diluted 1:25,000 in 5% nonfat milk–TBST, was added and incubated with the blot for at least 4 h. Goat anti-rabbit IgG AP-conjugated secondary antibody (Jackson ImmunoResearch) was added at a concentration of 1:25,000 and incubated at room temperature for 1 h. ECF detection and visualization were performed as previously described.

### Multilocus sequence typing (MLST).

Reaction mixtures (30 µl) were prepared in Thermo-Fast 96 ABgene PCR plates (Thermo Scientific) by combining 3 µl of 10× buffer, 2.5 µl of deoxynucleoside triphosphates (dNTPs) (2.5 mM), 18.5 µl distilled water (dH_2_O), 1.2 µl MgCl_2_, 2.5 µl of chromosomal DNA, 0.8 µl of both forward and reverse primers (10 µM; the primers are listed in Table 3), and 0.2 µl of Taq polymerase (Thermo). Plates were sealed with film, and then PCR was performed with the following parameters: initial denaturation at 95°C for 3 min, followed by 25 cycles of denaturation at 95°C for 15 s, annealing at 50°C for 30 s, and extension at 72°C for 30 s, with a final extension at 72°C for 10 min. DNA amplification was confirmed by agarose gel electrophoresis and visualized by exposure to UV light following ethidium bromide staining.

To prepare samples for sequencing, 2.5 µl of PCR product was added to a mixture of 2.5 µl of water and 1.5 µl of exonuclease I (0.25%; Affymetrix) and shrimp alkaline phosphatase (2.5%; Affymetrix) per well of a 96-well GeneMate FastPlate DNA sequencing plate (BioExpress). Reaction mixtures were incubated at 37°C for 30 min followed by a 5-min incubation at 95°C to inactivate enzymes. Primer (1 µl [2.5 µM]) was added to each well before the plates were sent to the University of Nebraska Medical Center (UNMC) core sequencing laboratory for Illumina HiSeq sequencing using an Applied Biosystems (ABI) 3730 48-capillary electrophoresis DNA analyzer.

### Determination of sequence types and genetic clusters.

The alleles at all 7 MLST loci were distinguished by sequence comparisons using the publically available *S. epidermidis* MLST database (http://sepidermidis.mlst.net/). Sequence types (STs) were assigned based on allelic profiles. Novel alleles and STs identified during the study were submitted to the database curator for number assignment. Sequences of 88 clinical isolates (44 high-shear and 44 low-shear strains) were assigned to genetic clusters (GCs) from the Bayesian clustering of the MLST database as reported by Tolo et al. (79).

### Generation of biofilm-enriched variants.

Methods were adapted from those reported by Christner et al. (36). Fifty-milliliter tissue culture flasks containing 5 ml TSB–10 µg/ml trimethoprim were inoculated with single colonies of the 1457 Δ*ica* mutant strain and grown statically at 37°C for 24 h. The flasks were then subjected to vortex mixing, the medium was removed, the flasks were washed with 5 ml fresh TSB, the medium was removed, and, finally, 5 ml fresh TSB–10 µg/ml trimethoprim was added and the flasks were incubated statically at 37°C for 2 to 4 days. The medium was removed, and the flasks were washed twice with 5 ml sterile PBS. The flask interiors were scraped and the contents plated on TSA–10 µg/ml trimethoprim and then incubated at 37°C for 24 h. Single colonies were patched onto TSA–10 µg/ml trimethoprim and tested for biofilm formation in the static assay as described previously.

### BioFlux biofilm formation and visualization.

Overnight cultures were diluted to an OD_600_ of 0.2. Channels of 24-well plates (Fluxion Biosciences Inc., South San Francisco, CA) were primed from outlet wells with 75% TSB at 10 dyne/cm^2^. To seed bacteria, 300 µl 75% TSB and 300 µl of diluted overnight cultures were added to outlet wells (final inoculum of OD_600_ = 0.1), and 310 µl 75% TSB was added to both inlet wells (A and B) to prevent backflow. Inocula were flowed from outlet wells at 2 dyne/cm^2^ for 4 s, and the bacteria were allowed to adhere for 1 h. TSB (75%) was supplied continuously at a shear rate of 0.8 dyne/cm^2^, and images were acquired every 5 min for the duration of the experiment. Image analysis and movie creation were performed using BioFlux Montage software.

### High-throughput sequencing and variant detection.

Sequencing was performed on a MiSeq platform (Illumina, San Diego, CA). For each sample, between 1.8 and 2.8 M paired-end reads (150-bp pairs) were obtained. The reads were aligned to the reference assembly of *Staphylococcus epidermidis* ATCC 12228 (NC_004461.1) using Bowtie2 (98). Alignments were processed with SAMtools (99) for format conversion and for the removal of putative PCR artifacts. Variant detection was then performed with SAMtools and VarScan2 (100). Finally, SNPEff (101) was employed to annotate detected variants.

### Statistical analyses.

Differences between high- and low-shear isolates with respect to the presence of the *ica* operon or *aap* were assessed separately by χ^2^ tests assuming equal distributions, with the null hypothesis that there was no difference in carriage of *icaADBC* or *aap*, respectively, between shear groups. To investigate associations between shear groups and biofilm phenotype, *agr* type, or genetic cluster, contingency tables were generated and analyzed by χ^2^ test for association, with the null hypotheses that there was no association between shear groups and biofilm phenotype, *agr* type, or genetic cluster, respectively (Microsoft Excel). Differences in *icaA* expression under conditions of slow and fast shear were assessed by Student’s *t* test.
